# Chinese medicinal formula Guanxin Shutong capsule protects the heart against oxidative stress and apoptosis induced by ischemic myocardial injury in rats

**DOI:** 10.3892/etm.2014.1540

**Published:** 2014-02-13

**Authors:** YANJUN CAO, XU HE, FENG LUI, ZHUANGZHUANG HUANG, YANMIN ZHANG

**Affiliations:** 1 School of Medicine, Xi’an Jiaotong University, Xi’an, Shaanxi 710061, P.R. China; 2 School of Pharmacy, Shaanxi University of Chinese Medicine, Xianyang, Shaanxi 712046, P.R. China

**Keywords:** Guanxin Shutong capsule, myocardial infarction, oxidative stress, NADPH oxidase

## Abstract

Guanxin Shutong capsule (GXSTC) is a Chinese medicinal formula that has been used clinically for the treatment of chest pain, depression, palpitation and cardiovascular diseases in China for almost 10 years. The aim of the present study was to investigate the protective mechanisms against oxidative stress and apoptosis that GXSTC exhibits in the hearts of rats with myocardial ischemia (MI). Infarct size and the levels of marker enzymes, including serum creatine kinase-isoenzyme (CK-MB), lactate dehydrogenase (LDH) and glutamate oxaloacetic transaminase (GOT), as well as the levels of nitric oxide (NO) and NO synthase (NOS) in the heart were measured by biochemical analysis assays. Levels of the antioxidants superoxide dismutase (SOD), catalase (CATA), and glutathione (GSH), and the oxidative stress marker malondialdehyde (MDA), were also determined. Following a 6-week period of ischemia, myocardial apoptosis, as well as the protein and mRNA expression of NADPH oxidase, was evaluated. Myocardial NADPH oxidase activity was measured by protein expression of p47phox and gp91phox using western blot analysis and mRNA expression of p22phox, p47phox, p67phox and gp91phox using reverse transcription polymerase chain reaction. The results showed that daily oral treatment of the rats with GXSTC reduced infarct size, myocardial apoptosis, the levels of serum MDA, LDH, CK-MB and GOT and heart GOT, and increased the activities of total SOD, CATA, NOS and the levels of NO and GSH compared with those in vehicle-treated MI model rats. Administration of GXSTC for 6 weeks also reduced the mRNA expression of the NADPH oxidase subunits p47phox and gp91phox protein, as well as the expression of Bax and caspase-3 proteins. By contrast, Bcl-2 protein expression increased. In conclusion, the results demonstrate that GXSTC attenuates myocardial injury via antioxidative and antiapoptotic effects.

## Introduction

Ischemic heart disease (IHD), which leads to myocardial infarction (MI), is a major clinical problem. Numerous patients continue to present with angina and myocardial ischemia (1). A pathological study has shown that ischemic apoptosis plays a critical role in acute MI (2) and apoptosis and necrosis have been reported to be associated with this clinical condition (3). In cardiac myocytes *in vitro*, the inhibition of NADPH oxidase reduces apoptosis (4). In previous years, studies have found that a number of traditional plants and their extracts have antioxidative effects on IHD (5,6).

IHD is the principal etiology for the development of congestive heart failure. Sustained ischemia causes several types of damage to cardiac tissues, including cardiomyocyte apoptosis (7). Heart failure following MI is a common clinical problem and has a poor prognosis. It has been demonstrated that free radicals are involved in the pathogenesis of heart failure, subsequent to MI. Changes in myocardial antioxidant levels, as well as oxidative stress, have been observed in the surviving myocardium of rats subjected to MI (8,9). The early stages of MI are accompanied by a significant increase in oxidative stress and lipid peroxidation, with significant reductions in glutathione (GSH), catalase (CATA) and superoxide dismutase (SOD) levels. The mechanism of action of Bcl-2 in the prevention of apoptosis has also been hypothesized to be mediated by an antioxidant pathway. Reactive oxygen species (ROS) have been implicated in myocardial hypertrophy, apoptosis, contractile dysfunction and fibrosis (3).

Traditional Chinese medicine has been shown to have specific prospective therapeutic effects on IHD. Guanxin Shutong capsule (GXSTC) is a widely used Chinese medicinal formula, which is clinically administered for palpitations, restlessness, short breath, fatigue, dizziness and chest pain, promoting blood circulation, removing blood stasis (9) and protecting against cardiovascular diseases (10,11). GXSTC contains five traditional Chinese drugs: Choerospondiatis, *Salvia miltiorrhiza*, lilac, borneol and tabasheer. Studies have indicated that lilac possesses antioxidative properties (10) and choerospondiatis and *Salvia miltiorrhiza* exhibit anti-ischemic therapeutic effects (12), as well as the ability to scavenge active oxygen free radicals (13).

Experimental and clinical studies have indicated that GXSTC has various cardiovascular pharmacological effects. However, there is little information available concerning the antioxidative mechanisms of GXSTC. Therefore, the purpose of the present study was to evaluate whether orally administered CXSTC protects the heart against oxidative stress and apoptosis in rats with MI.

## Materials and methods

### Surgical preparation of the animals

Experimental procedures and protocols on animals were approved by the Ethics Committee of Xi’an Jiaotong University for Animal Research (Xi’an, China). Male Wistar rats, weighing 290–310 g (purchased from school of medicine, Xi’an Jiaotong University, Xi’an, China), were anesthetized by the intraperitoneal injection of 35 mg/kg pentobarbital sodium. Following intratracheal intubation, a left thoracotomy was performed under volume-controlled mechanical ventilation. The heart was raised from the thorax and a ligature with a 6-0 Prolene suture was placed around the proximal left anterior descending coronary artery. The chest was then closed. The same surgical procedures were performed in sham-operated rats, with the exception that the suture around the coronary artery was not tied. Samples were collected from the marginal region around the infarction as samples of infarcted sites.

GXSTC consists of five traditional Chinese drugs: 57.1% choerospondiatis, 28.6% *Salvia miltiorrhiza*, 7.1% lilac, 3.6% borneol and 3.6% tabasheer. GXSTC was provided by Buchang Pharmaceuticals (batch no. 20120125; Xi’an, China). Following removal of the capsules, the Guanxin Shutong powder was dissolved in 40 mg/ml aseptic sodium carboxymethylcellulose (CMC-Na; 0.5%). Within 24 h after modeling, the solution was administered intragastrically each day at 0.2 g/kg GXSTC for the high dosage group (GXSTCH), 0.1 g/kg GXSTC for the low dosage group (GXSTCL) and 10 ml/kg CMC-Na for the sham surgery and MI + vehicle groups (8 rats for each group). After 6 weeks of treatment, 24-h urine samples and blood were harvested. Following decapitation, the hearts were removed, snap-frozen in liquid nitrogen and stored at −80°C until required for processing for protein or mRNA extraction.

### Determination of the infarct size

The heart tissue was washed with PBS three times. Sections were sliced and then incubated for 10 min in nitrotetrazolium blue chloride for pathological examination. Photographs were captured and the infarct size was measured with Image-Pro Plus software (MediaCybernetics, Inc., Rockville, MD, USA). Ischemic and left ventricular areas were determined in five slices of each heart tissue sample. The ischemic risk area ratio was defined as a percentage and calculated as follows: Total ischemic area/total left ventricular area × 100.

### Antioxidant assays

To determine the total SOD and CATA activities, as well as the malondialdehyde (MDA) and GSH levels, blood was sampled from the abdominal aorta and serum was obtained following centrifugation at 3,000 × g for 10 min. The MDA and GSH levels, and SOD and CATA activities, were measured spectrophotometrically using diagnostic kits (Jiancheng Bioengineering Institute, Nanjing, China), according to the manufacturer’s instructions.

### Determination of serum creatine kinase-isoenzyme (CK-MB), serum lactate dehydrogenase (LDH) and serum glutamate oxaloacetic transaminase (SGOT)

Blood was sampled from the abdominal aorta and serum was obtained by centrifugation at 3,000 × g for 10 min. CK-MB, LDH and SGOT levels were determined spectrophotometrically at 660, 450 and 510 nm, respectively, using diagnostic kits (Jiancheng Bioengineering Institute), according to the manufacturer’s instructions.

### Determination of heart SOD, GOT, nitric oxide (NO) and NO synthase (NOS)

Heart tissue was homogenized in ice-cold 0.9% saline solution and centrifuged at 600 × g for 10 min. The supernatant was used to determine SOD, GOT, NO and NOS levels, which were measured spectrophotometrically using diagnostic kits (Jiancheng Bioengineering Institute) according to the manufacturer’s instructions.

### mRNA expression of NADPH oxidase subunits

The mRNA expression levels of p22phox, p47phox, gp91phox and p67phox in the renal cortical tissues were quantitatively analyzed by reverse transcription polymerase chain reaction (RT-PCR), as described previously (14,15). Primer sequences for the analysis of p22phox, p47phox, p67phox, gp91phox and GAPDH mRNA are shown in Table I. GAPDH was measured as an invariant housekeeping gene for an internal control. Amplified cDNA bands were detected by Goldview staining (RuiTaibio, Beijing, China) and the quantities of mRNA were evaluated using the Gene Genius imaging system (Syngene, Cambridge, UK).

### Determination of Bcl-2, Bax, caspase-3, gp91phox and p47phox protein expression

Rabbit monoclonal antibodies against Bcl-2, Bax, p47phox (Santa Cruz Biotechnology, Inc., Santa Cruz, CA, USA), gp91phox and caspase-3 (Epitomics, Burlingame, CA, USA), were used to determine the protein expression levels of gp91phox, p47phox, Bcl-2 and Bax by western blot analysis, as described previously (16,17).

### Statistical analysis

All data are expressed as mean ± SD. Statistical analysis was performed by Student’s t-test or one-way ANOVA, followed by a Tukey’s multiple comparison test. P<0.05 was considered to indicate a statistically significant difference.

## Results

### Effect of GXSTC on infarct size

The ischemic risk area ratio was 33.93±6.56% in the MI + vehicle group. Treatment with GXSTC at doses of 0.1 and 0.2 g/kg body weight resulted in dose-dependent reductions in infarct size, with ischemic risk area ratios of 23.45±4.67 and 17.82±5.89%, respectively. Significant differences were observed in the ratios between the MI + vehicle and GXSTCL and GXSTCH groups (Table II).

### Antioxidant assays

**Figure 1:**
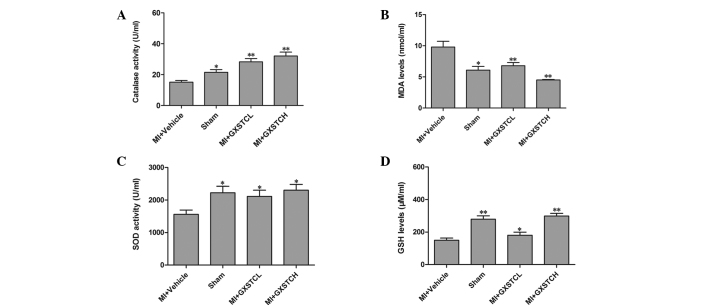
Effect of GXSTC on the serum levels of (A) CATA, (B) MDA, (C) SOD and (D) GSH. Sham (sham-operated control; n=12), MI + vehicle (orally administered vehicle; n=12), MI + GXSTCH (orally administered 0.2 g/kg GXSTC; n=12) and MI + GXSTCL (orally administered 0.1 g/kg GXSTC; n=12). ^*^P<0.05 and ^**^P<0.01, vs. MI + vehicle. GXSTC, Guanxin Shutong capsule; CATA, catalase; MDA, malondialdehyde; SOD, superoxide dismutase; GSH, glutathione; MI, myocardial infarction.

The serum SOD, GSH and CATA levels of the MI + vehicle group were significantly decreased (1,560.90±130.02, 149.80±18.95 and 15.18±1.16 U/ml, respectively), while the serum MDA level was significantly increased (9.8 ± 0.93 nmol/ml) compared with the corresponding levels in the sham-operated rats (Fig. 1). The GXSTCL and GXSTCH groups showed significantly reduced serum levels of MDA (6.86±0.58 and 4.59±0.15 nmol/ml, respectively). By contrast, the levels of SOD, GSH and CATA were increased (to 2,110.8.56±190.94 and 2,300.92±180.49 U/ml; 180.58±18.77 and 298.37±16.54 mg/l; and 28.34±2.19 and 32.35±2.56 U/ml, respectively) (P<0.05; Fig. 1).

### Effect of GXSTC on the serum levels of CK-MB, LDH and GOT

**Figure 2:**
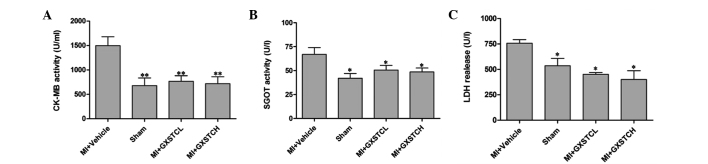
Effect of GXSTC on (A) serum CK-MB (B) SGOT and (C) serum LDH. Sham (sham-operated control; n=12), MI + vehicle (orally administered vehicle; n=12), MI + GXSTCH (orally administered 0.2 g/kg GXSTC; n=12) and MI + GXSTCL (orally administered 0.1 g/kg GXSTC; n=12). ^*^P<0.05 and ^**^P<0.01, vs. MI + vehicle. GXSTC, Guanxin Shutong capsule; LDH, lactate dehydrogenase; CK-MB, creatine kinase-isoenzyme; SGOT, serum glutamate oxaloacetic transaminase; MI, myocardial infarction.

The serum levels of CK-MB, LDH and GOT in the MI + vehicle group were increased significantly (1,499.43±180.30, 756.97±35.64 and 66.99±7.06 U/ml, respectively) compared with those in the sham group (Fig. 2). GXSTC at doses of 0.1 and 0.2 g/kg body weight significantly reduced the serum level of CK-MB to 767.85±113.0 and 719.51±140.11 U/ml, respectively, and of LDH to 451.20±18.18 and 400.48±86.24 U/ml, respectively (P<0.05; Fig. 2). In addition, GXSTC at both doses significantly decreased SGOT activity.

### Effect of GXSTC on the levels of SOD, GOT, NO and NOS in the heart

**Figure 3:**
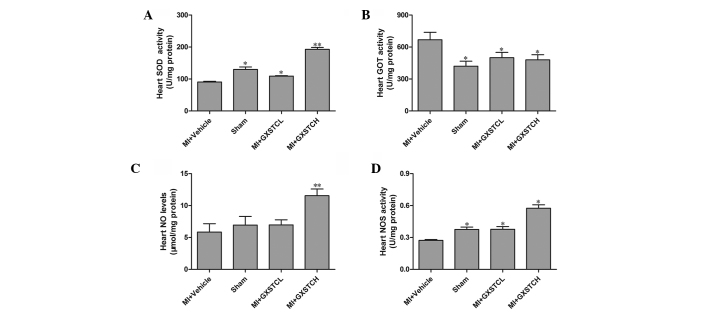
Effect of GXSTC on the levels of (A) SOD, (B) GOT, (C) NO and (D) NOS in the heart. Sham (sham-operated control; n=12), MI + vehicle (orally administered vehicle; n=12), MI + GXSTCH (orally administered 0.2 g/kg GXSTC; n=12) and MI + GXSTCL (orally administered 0.1 g/kg GXSTC; n=12). ^*^P<0.05 and ^**^P<0.01, vs. MI + vehicle. GXSTC, Guanxin Shutong capsule; SOD, superoxide dismutase; GOT, glutamate oxaloacetic transaminase; NO, nitric oxide; NOS, nitric oxide synthase; MI, myocardial infarction.

The SOD, NO and NOS levels in the hearts of the MI + vehicle group were significantly decreased whereas the levels of GOT were significantly increased compared with those in the sham-operated rats. In the CXSTCH group, the levels of SOD, NOS and NO (192.81 ± 5.91 and 0.58 ± 0.03 U/mg protein and 11.55 ± 1.05 μg/mg protein, respectively) were significantly increased compared with those in the MI + vehicle group (90.74 ± 2.08 and 0.27 ± 0.01 U/mg protein and 5.84 ± 1.31 μg/mg protein, respectively), whereas the levels of GOT were significantly decreased (from 668.99 ± 70.06 to 480.85 ±47.11 U/mg protein) (P<0.05, Fig. 3). In addition, the GXSTCL group also showed significantly decreased levels of GOT and increased levels of SOD, NO and NOS in the heart (Fig. 3).

### Effect of GXSTC on the mRNA expression of NADPH oxidase subunits

**Figure 4:**
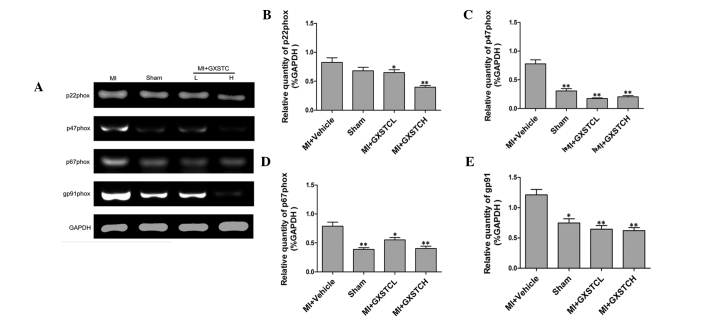
Effect of GXSTC on p22phox, p47phox, p67phox and gp91phox mRNA expression in the heart. (A) Bands correspond to p47phox, p22phox, p67phox, gp91phox and GAPDH. Results of (B) p22phox, (C) p47phox, (D) p67phox and (E) gp91phox were quantified by densitometry analysis of the bands from (A) and then normalized against GAPDH in the heart tissue. Sham (sham-operated control; n =12), MI + vehicle (orally administered vehicle; n=12), MI + GXSTCH (orally administered 0.2 g/kg GXSTC; n=12) and MI + GXSTCL (orally 0.1 g/kg administered GXSTC; n=12). ^*^P<0.05 and ^**^P<0.01, vs. MI + vehicle. GXSTC, Guanxin Shutong capsule; MI, myocardial infarction.

MI model rats showed significantly higher p22phox, p47phox, p67phox and gp91phox mRNA expression levels compared with sham-operated rats. Treatment with GXSTC (0.1 and 0.2 g/kg/day) significantly decreased p22phox, p47phox, p67phox and gp91phox mRNA expression (Fig. 4).

### Effect of GXSTC on Bcl-2, Bax and caspase-3 proteins

**Figure 5:**
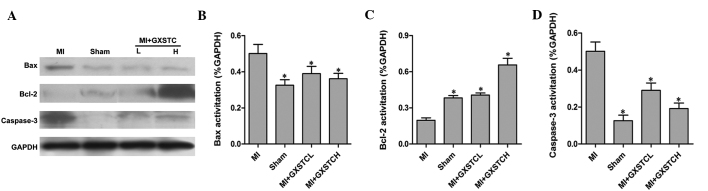
Effect of GXSTC on apoptosis-associated protein expression in all treated groups. (A) Bands correspond to Bcl-2, Bax, caspase-3 and GAPDH. Results of (B) Bax, (C) Bcl-2 and (D) caspase-3 were quantified by densitometry analysis of the bands from (A) and then normalized against GAPDH in the heart tissue. Results were obtained from three independent experiments performed in triplicate. ^*^P<0.05 and ^**^P<0.01, vs. MI + vehicle. GXSTC, Guanxin Shutong capsule; MI, myocardial infarction.

The expression of the apoptosis-related proteins Bcl-2, Bax and caspase-3 was studied by western blot analysis. Following the induction of MI, the expression of Bax and caspase-3 markedly increased, whereas the expression of Bcl-2 decreased (Fig. 5). The MI-induced changes in protein levels were significantly reversed by GXSTC. These results indicated that GXSTC may modulate MI-induced apoptosis via Bcl-2, Bax and caspase-3 proteins (Fig. 5).

### Effect of GXSTC on p47phox and gp91phox proteins

**Figure 6:**
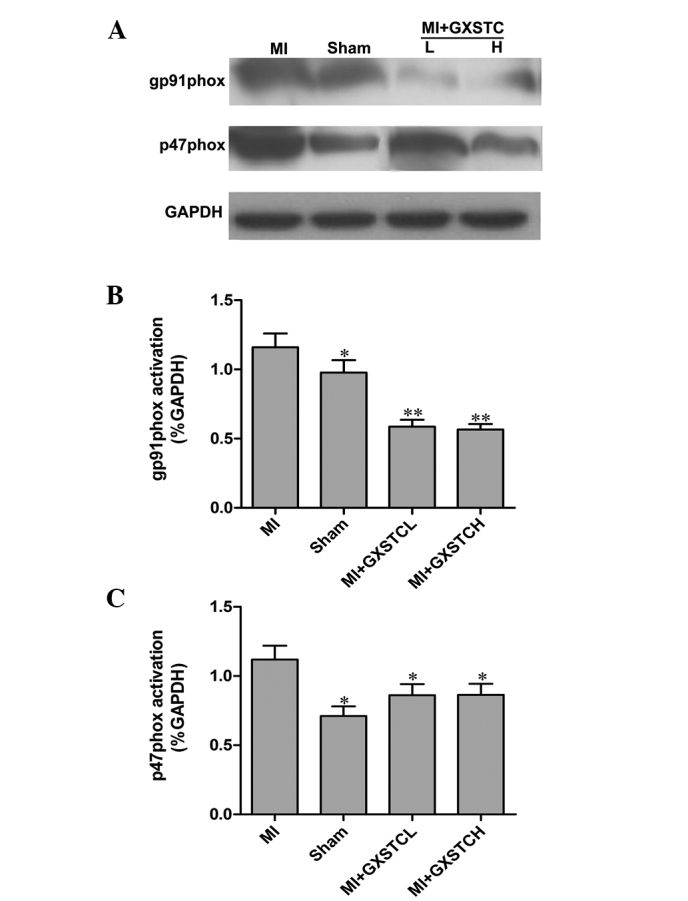
Effect of GXSTC on the NADPH oxidase subunits, p47phox and gp91phox, in heart tissue. (A) Bands correspond to p47phox, gp91phox and GAPDH. (B) gp91phox and (C) p47phox were quantified by densitometric analysis of the bands from (A) and then normalized against GAPDH protein. Results were obtained from three independent experiments performed in triplicate. ^*^P<0.05 and ^**^P<0.01, vs. MI + vehicle. GXSTC, Guanxin Shutong capsule; MI, myocardial infarction.

Western blot analysis was performed on heart homogenates to determine the protein expression levels of the NADPH oxidase subunits gp91phox and p47phox. As shown in Fig. 6, the protein levels of gp91phox and p47phox decreased significantly in the hearts of rats with MI following treatment with GXSTC.

## Discussion

GXSTC has been reported to be effective in treating MI in animal models and cardiovascular diseases in humans (18). Results of the present study show that GXSTC dose-dependently reduced the size of the infarct caused by ischemic injuries in rats, which is the most reliable indicator of myocardial protection. In addition, it was demonstrated that GXSTC effectively reduced CK-MB, LHD, MDA and SGOT levels in rats with MI, as well as the protein and mRNA expression of NADPH oxidase subunits, but enhanced CATA and SOD activities and GSH levels. Furthermore, protein expression of proapoptotic Bax and caspase-3 were significantly downregulated, while the antiapoptotic protein, Bcl-2, was upregulated in the GXSTC treated groups when compared with the MI + vehicle group.

LDH and CK, which are released into plasma from myocardial tissues, are representative of cardiac cellular damage during MI (19). In the present study, GXSTC significantly increased the serum levels of SOD, indicating that GXSTC possesses a potent antioxidant property. In addition, it was observed that the level of LDH, a biochemical indicator of cellular damage, was also dose-dependently decreased. Previous studies have shown that acute myocardial ischemia generates numerous free radicals, causing damage to cellular membranes as a result of lipid peroxidation. MDA, formed by the breakdown of lipid peroxides, is often used to quantify the extent of lipid peroxidation (20), whereas serum CK-MB is a cardiac-specific marker of acute MI or an indicator for myocardial tissue injury. The present study demonstrated that GXSTC significantly prevented an increase of serum MDA and CK-MB levels caused by acute ischemic injury, indicating that GXSTC may exert its protective effect against myocardial ischemic injury by reducing lipid peroxidation. Choerospondiatis and *Salvia miltiorrhiza* contain polyphenolic compounds with antioxidant properties, which are considered to provide the pharmacological efficacy against heart ischemia (21). The mechanisms of action of choerospondiatis and *Salvia miltiorrhiza* are distinct and complementary when combined together. Therefore, GXSTC may be synergistic and more effective than the individual components.

MDA, the degradation product of oxygen-derived free radicals and lipid oxidation, reflects the damage caused by ROS (22). Studies on the antioxidant system have shown that changes in SOD activity and MDA levels are always negatively correlated (23). In the present study, GXSTC also increased the serum levels of SOD when administered at doses of 0.1 and 0.2 g/kg/day. However, MDA production was low which implied that the formula may affect the level of endogenous antioxidants and/or oxidative stress. One possible explanation is that the elevated SOD activities resulted in the scavenging of excessive ROS and attenuated lipid peroxidation. GSH is an important cellular reductant that offers protection against free radicals, peroxides and toxic compounds, which is reformed from glutathione disulfide (24). The results showed that administration of GXSTC caused a significant increase in GSH levels, which may be due to the compounds present in GXSTC.

Previous animal studies and clinical observations have indicated that the balance between antiapoptotic Bcl-2 and proapoptotic Bax and caspase-3 proteins plays a major role in regulating apoptosis (25,26). Overexpression of Bax and caspase-3 in cells leads to apoptotic death in response to apoptosis signals. In contrast, overexpression of Bcl-2 inhibits apoptosis and decreases the ROS formation and lipid peroxidation initiated by various stimuli (26). In pathological conditions, including MI, diabetes and stroke, the production of free radicals may override the scavenging effects of antioxidants, leading to oxidative stress (27). Thus, targeting myocardial apoptosis is a reasonable therapeutic strategy for reducing the risk of ischemic injury. It had been indicated that Bcl-2 inhibits the apoptosis induced by ROS through an antioxidative pathway (25). Analysis of the expression and regulation of Bcl-2 family proteins may provide insight as to their role in ischemia-induced apoptosis and stress adaptation. The present study observed that GXSTC treatment increased Bcl-2 protein expression during MI, as determined by western blot analysis.

Systematic reviews have shown that oxidative stress may powerfully induce programmed cell death. ROS are implicated in myocardial hypertrophy, apoptosis, contractile dysfunction and fibrosis (28). Increases in NADPH oxidase activity, oxidative stress and myocyte apoptosis have been observed concurrently in failing hearts (29). In cardiac myocytes, the *in vitro* inhibition of NADPH oxidase reduces apoptosis. NADPH oxidase subunits, gp91phox, p22phox, p40phox, p47phox, p67phox and rac1, have been found to be expressed in endothelial cells, vascular smooth muscle cells, cardiomyocytes and fibroblasts (30). The increased expression of NADPH oxidase subunits, gp91phox and p22phox, has been associated with lipid peroxidation levels following acute MI in rats (31). The present study shows that GXSTC is able to reduce the increased protein and mRNA expression of NADPH oxidase subunits and oxidative stress associated with remote infarct myocardium in rats. In response to the treatment, the animals’ natural defensive system was activated to cope with the unwanted and toxic species, including increased production of SOD, GSH and CATA. The present study indicates that orally administered GXSTC possesses antioxidative properties. The results are consistent with those of a previous study demonstrating that NADPH oxidase activation mediates ROS production in cardiac hypertrophy and failure (3,30), which is also implicated in myocyte apoptosis.

In conclusion, the present study demonstrates that GXSTC has significant cardioprotective effects against ischemic myocardial injury in rats, which is likely to be due to its antioxidant and antiapoptotic properties. Therefore, GXSTC may be used as an effective and promising medicine for the prophylaxis and treatment of IHD.

## Figures and Tables

**Table I:** Primers used for p22phox, p47phox, p67phox, gp91phox and GAPDH.

mRNA	Primer	Sequence, 5′-3′
p22phox	Sense	ATGGAGCGGTGTGGACAGAAG
	Antisense	CGGACAGCAGTAAGTGGAGGAC
p47phox	Sense	CCATCATCCTTCAGACCTATCG
	Antisense	AACCACCAGCCACTCTCG
p67phox	Sense	CGTGTGTTGTTTGGCTTTGTG
	Antisense	CTGAGGCTGCGACTGAGG
gp91phox	Sense	TAGCATCCATATCCGCATTG
	Antisense	CTAACATCACCACCTCATAGC
GAPDH	Sense	AGTGGCAAAGTGGAGATT
	Antisense	GTGGAGTCATACTGGAACA

**Table II:** Effect of GXSTC on the myocardial infarct size following ligature of the left anterior descending coronary artery.

Group	Infarct size, cm^2^	Left ventricular areas, cm^2^	Infarct-to-left ventricular areas, %
MI + vehicle	1.33±0.29	2.59±0.19	33.93±6.56
Sham	0	3.13±0.21	0
GXSTCL	0.87±0.09	2.84±0.13	23.45±4.67^a^
GXSTCH	0.59±0.06	2.72±0.26	17.82±5.89^b^

Data are presented as mean ± SD (n=12 for each group).

a P<0.05 and

b P<0.01, vs. MI + vehicle.

GXSTC, Guanxin Shutong capsule; GXSTCL, 0.1 g/kg body weight GXSTC; GXSTCL, 0.2 g/kg body weight GXSTC.
